# Reduced odds of diabetes associated with high plasma salivary α-amylase activity in Qatari women: a cross-sectional study

**DOI:** 10.1038/s41598-021-90977-y

**Published:** 2021-06-01

**Authors:** Neyla S. Al-Akl, Richard Ian Thompson, Abdelilah Arredouani

**Affiliations:** 1 Diabetes Research Center, Qatar Biomedical Research Institute (QBRI), Qatar Foundation, Hamad Bin Khalifa University (HBKU), PO Box: 34110, Doha, Qatar; 2 College of Health and Life Sciences, Qatar Foundation, Hamad Bin Khalifa University (HBKU), Doha, Qatar

**Keywords:** Predictive markers, Type 2 diabetes, Gene expression

## Abstract

The association of salivary α-amylase activity (SAA) activity or low copy number of its coding gene AMY1 with diabetes remains controversial. We aimed to reinvestigate the association of these factors with diabetes in Qatar, where diabetes prevalence is about 16%. We obtained cross-sectional data of 929 Qataris (age > 18 years) from the Qatar Biobank. We estimated AMY1 copy number variants (CNV) from whole-genome data, and quantified the SAA activity in plasma (pSAA). We used adjusted logistic regression to examine the association between pSAA activity or AMY1 CNV and diabetes odds. We found a significant association between high pSAA activity, but not AMY1 CNV, and reduced odds of diabetes in Qatari women. The OR per pSAA activity unit was 0.95 [95% CI 0.92, 0.98] (p = 0.002) (pSAA activity range: 4.7 U/L to 65 U/L) in women. The association is driven largely by the highest levels of pSAA activity. The probability of having diabetes was significantly lower in the fifth pSAA activity quintile relative to the first (0.21 ± 0.03 (Q1) versus 0.82 ± 0.02 (Q5)), resulting in significantly reduced diabetes prevalence in Q5 in women. Our study indicates a beneficial effect of high pSAA activity, but not AMY1 CN, on diabetes odds in Qatari women, and suggests pSAA activity levels as a potential marker to predict future diabetes in Qatari women.

## Introduction

The salivary α-amylase (SAA) represents at least 50% of total saliva protein content^1^. It initiates the digestion in the mouth of large α-linked polysaccharides, such as starch or glycogen, to yield a mixture of di-saccharides, tri-saccharides, and some glucose^2^. In humans, the AMY1 gene codes for SAA and the copy number variations (CNVs) in this gene influences the production of the enzyme^3^. AMY1 is one of the most variable loci in the human genome, ranging from 2 to 20 diploid copies^3–6^. Both the saliva and serum levels of SAA correlate positively with the number of copies of the AMY1 gene^3–5,7^. Variability in both AMY1 CNVs and SAA protein levels occurs among human populations, with those traditionally consuming starch-rich diets showing higher AMY1 gene CNV and SAA levels than those consuming low-starch diets^3,8^. Several population studies on both adults and school children identified a negative correlation between AMY1 gene CN and common obesity. Low AMY1 gene CN was associated with an increased body mass index (BMI) and waist circumference^5,9–14^. However, other studies reported no association^15–17^ or a positive association between the AMY1 gene CN and BMI^18^.

Furthermore, AMY1 CN inversely correlates with high insulin resistance^19^, as well as with increased cardiovascular disease risk and inflammation in overweight or obese adults^20^. Also, low SAA activity negatively associates with a behavioral preference for foods high in sugar^21^, which may explain the predisposition to obesity^5^. Furthermore, numerous nutritional studies have highlighted the role of SAA in the digestion of dietary starch and have underscored its significance for post-prandial glycaemia. Given the production of simple sugars from starch upon amylase action, it was anticipated that individuals with high copies of AMY1 would display higher post-prandial glycaemia after starch ingestion, compared to low copies AMY1 counterparts. Paradoxically, however, some recent studies reported the opposite, i.e. higher post-prandial glycaemia in individuals with low AMY1 gene CN in response to starch ingestion but not to glucose or maltose ingestion^7,22–24^. These findings suggest that people with low pSAA activity might be at greater risk of glucose intolerance if they chronically consume starch-rich foods^23,24^. Altogether, these studies show that there exists a genetic link between carbohydrate metabolism and glucose homeostasis and that SAA may play a role in this link. Moreover, the observations above suggest that the pSAA activity and AMY1 CN could serve as potential markers to predict metabolic disorders, particularly in populations where starch is a staple food.

In most of the studies mentioned above, the relationship between SAA and metabolic disorders was suggested by investigating the association of the different metabolic traits with the AMY1 CNV. Different techniques were used to estimate AMY1 CNV in those studies resulting in contradicting results. Moreover, a recent study demonstrated that AMY1 CNV does not explain the majority of the observed variation in expression and activity between individuals^6^. The same study also showed that individuals with even and odd AMY1 CNV showed different, though not statistically significant (= 0.052), relationships between the AMY1 CNV and the SAA expression levels.

This observation, combined with the fact that SAA activity is essentially the end-product of the AMY1 gene, and is the factor which has the most significant effect on the degradation of polysaccharides, prompted us to investigate and compare the association between the odds of diabetes and plasma SAA activity or AMY1 CNV in adults in Qatar. The Middle East in general, and Qatar in particular, is one of the regions most-hit with diabetes, and starch, in the form of rice, is a staple food.

## Results

### General characteristics of the study participants

**Figure 1:**
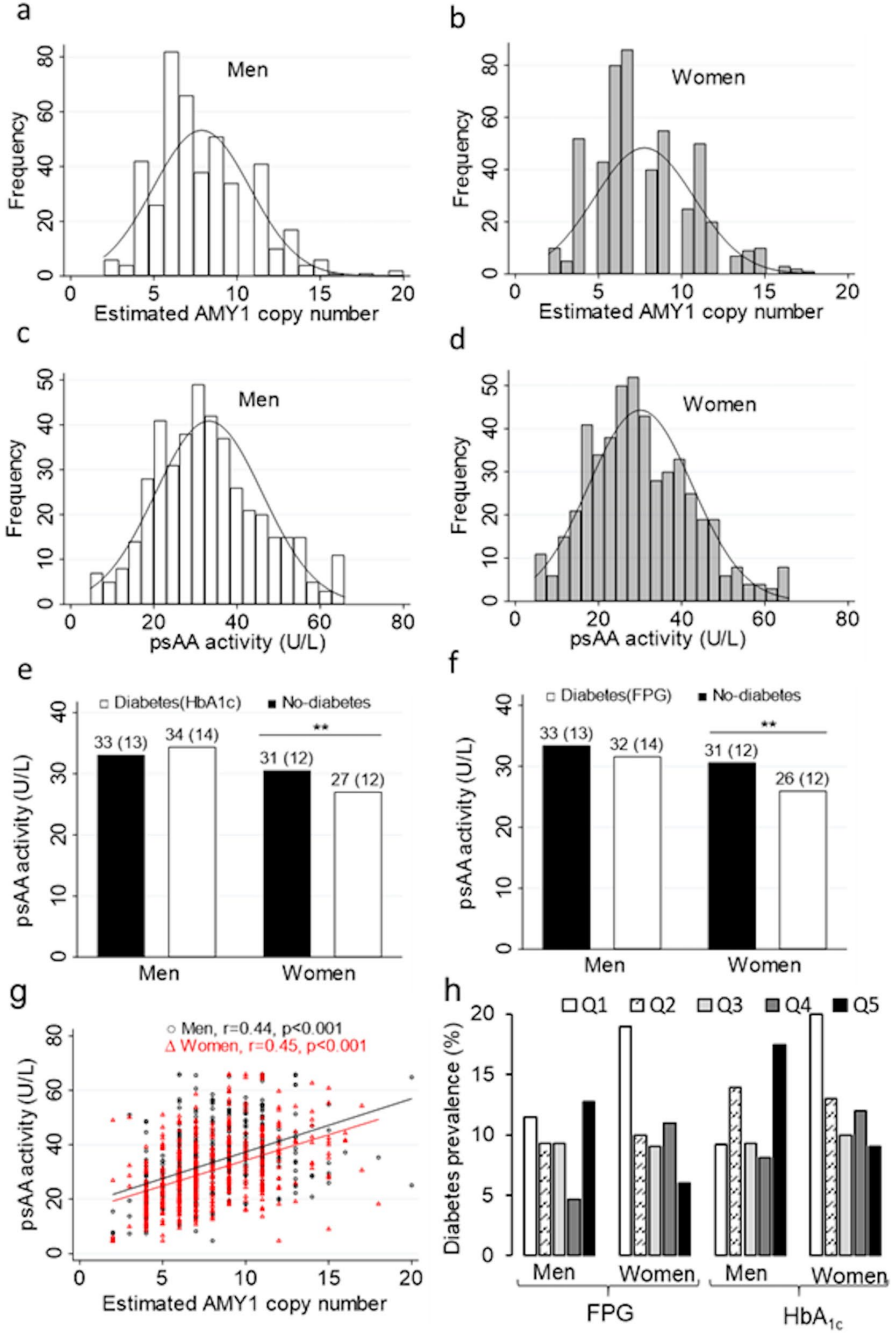
Distribution by gender of AMY1 CN (**a,b**), and pSAA activity (**c,d**). Comparison of mean pSAA activity in women and men with and without diabetes (**e,f**). Correlation between AMY1 CN and pSAA activity in men (black circles) and women (red triangles) (**g**) the straight lines represent the line of best fit for men (black) and women (red). Prevalence of diabetes by quintiles of pSAA activity (**h**). Data in (**e,f**) are mean ± SD. *And **significant at p < 5% and p < 1%, respectively.

Table 1 shows the general characteristics of the participants (53.6% women). The mean age was 39 ± 11 for men (n = 431) and 39.9 ± 13 years for women (n = 498). There was no significant gender difference in diabetes prevalence, whether we use HbA1c or FPG cutoff to define diabetes. Obesity was significantly higher in women (p = 0.01), while men were significantly more overweight (p = 0.02). BMI was not substantially different between the sexes. The number of copies of the AMY1 gene ranged from 2 to 20 copies (Fig. 1a,b) The plasma SAA activity (pSAA activity) ranged from 4.73 to 65.82 U/L (Fig. 1c,d). The median and mean pSAA activity were significantly higher in men (Table 1). There were no significant gender differences in mean or median AMY1 CNV, nor in the glycemic parameters tested, including FPG, HbA_1c_, insulin, HOMA-IR, and HOMA-β.

**Table 1:** General and clinical characteristics of the participants by gender.

Variables	Male (n = 431)	Female (n = 498)	*p*
Age (years)	39.0 (11.3)	39.9 (12.9)	0.25
AMY1 CNV (median)	7	7	0.47
AMY1 CNV (mean)	7.9 (2.9)	7.7 (3)	0.56
pSAA activity (U/L) (mean)	33.2 (12.8)	30.08 (12.4)	< 0.001***
pSAA activity (U/L) (median)	32	29	< 0.001***
Obesity %	38	46.4	0.01**
Overweight %	38	31	0.02*
Diabetes % (HbA_1c_)	11.6	12.8	0.56
Diabetes % (FPG)	9.5	11	0.44
BMI (kg/m^2^)	29.1 (5.5)	29.6 (6.3)	0.21
FPG (mmol/L)	5.7 (1.7)	5.6 (1.8)	0.50
HbA_1c_ (mmol/mol)	38 (11)	39 (11)	0.19
HbA_1c_ (%)	5.6(1.0)	5.7 (1.0)	0.18
Insulin (mU/mL)	10.9 (6.5)	10.7 (7.1)	0.58
HOMA-IR	2.8 (1.9)	2.8 (2.3)	0.80
HOMA-b	117.2 (68.1)	119.6 (70.7)	0.61

*CNV* copy number variations, *pSAA activity* plasma salivary alpha amylase activity, *BMI* body mass index, *FPG* fasting plasma glucose, *HbA*_*1c*_ glycated hemoglobin, *HOMA-IR* homeostatic model assessment of insulin resistance, *HOMA-b* homeostatic model assessment of beta cell function.

### Correlation between plasma SAA activity and AMY1 gene CNV

In both sexes, 90% of the individuals had AMY1 CNV between 4 and 12. There was a significant but moderate positive correlation between AMY1 CNV and pSAA activity in both sexes (r = 0.44; p < 0.001 for men, and r = 0.45; p < 0.001 for women) (Fig. 1g). Linear regression analysis adjusted for age showed that each additional AMY1 copy increased the pSAA activity by 1.97 U/L ([95% CI 1.6–2.3]) in men, and by 1.88 U/L ([95% CI 1.5–2.2]) in women and explaining only 20% of the pSAA activity variation in both sexes. The mean AMY1 CNV was not significantly different between individuals with and without diabetes in both sexes (Table 2). Interestingly, however, mean pSAA activity was significantly lower in women with diabetes, and not in men (Fig. 1e,f, Table 2).

**Table 2:** Association between pSAA activity or AMY1 CN and the odds of having diabetes in men and women.

Predictor variables	Men (n = 431)					Women (n = 498)				
	Diabetes based of HbA1c%									
	Cases (n = 50)	Controls (n = 381)	*p*	OR (95% CI)	*p*	Cases (n = 64)	Controls (n = 434)	*p*	OR (95% CI)	*p*
pSAA activity (U/L)	34.35 (14.07)	33.06 (12.70)	0.50	1.00 (0.98, 1.03)	0.45	30.5 (12.42)	27.01 (12.36)	**0.03**	0.95 (0.92, 0.98)	**0.002**
AMY1 CN	7.9 (2.8)	7.8 (2.9)	0.89	1.04 (0.93, 1.15)	0.43	7.5 (2.8)	77.8 (3)	0.49	0.98 (0.81, 1.01)	0.09

Predictor variables	Diabetes based of fasting plasma glucose									
	Cases (n = 381)	Controls (n = 50)	*p*	OR (95% CI)	*p*	Cases (n = 55)	Controls (n = 434)	*p*	OR (95% CI)	*p*
pSAA activity (U/L)	31.58 (14.18)	33.38 (12.72)	0.39	0.99 (0.96, 1.01	0.55	25.9 (12.16)	30.60 (12.41)	**0.009**	0.95 (0.92, 0.98)	**0.001**
AMY1 CN	7.8 (3.2)	7.8 (2.9)	0.47	1.03 (0.92, 1.15)	0.57	7.4 (2.8)	7.8 (3)	0.48	0.91 (0.81, 1.02)	0.12

pSAA activity and AMY CNV values are means and (SD). The table includes the logistic regression analysis adjusted for age and BMI. Cases are the individuals with diabetes (based on HbA1c or FPG), while controls are the individuals without diabetes.

The significant p values are shown in bold.

### Association of pSAA activity and AMY1 CN with odds of diabetes

Using a logistic regression model adjusted for age and BMI, we assessed the association between pSAA activity or AMY1 CNN and the odds of having diabetes in a binary case–control (diabetes/no-diabetes) framework. Only in women did we find a significant association between high pSAA activity and reduced odds of having diabetes, whether we used FPG or HbA_1c_ cutoff to define diabetes (OR per one pSAA activity unit 0.95 [95% CI 0.92, 0.98], p = 0.002; bearing in mind that the pSAA activity ranges from 4.7 to 69 U/L in our population) (Table 2). Interestingly, however, no significant association was found between AMY1 CNV and the odds of having diabetes (Table 2).

**Figure 2:**
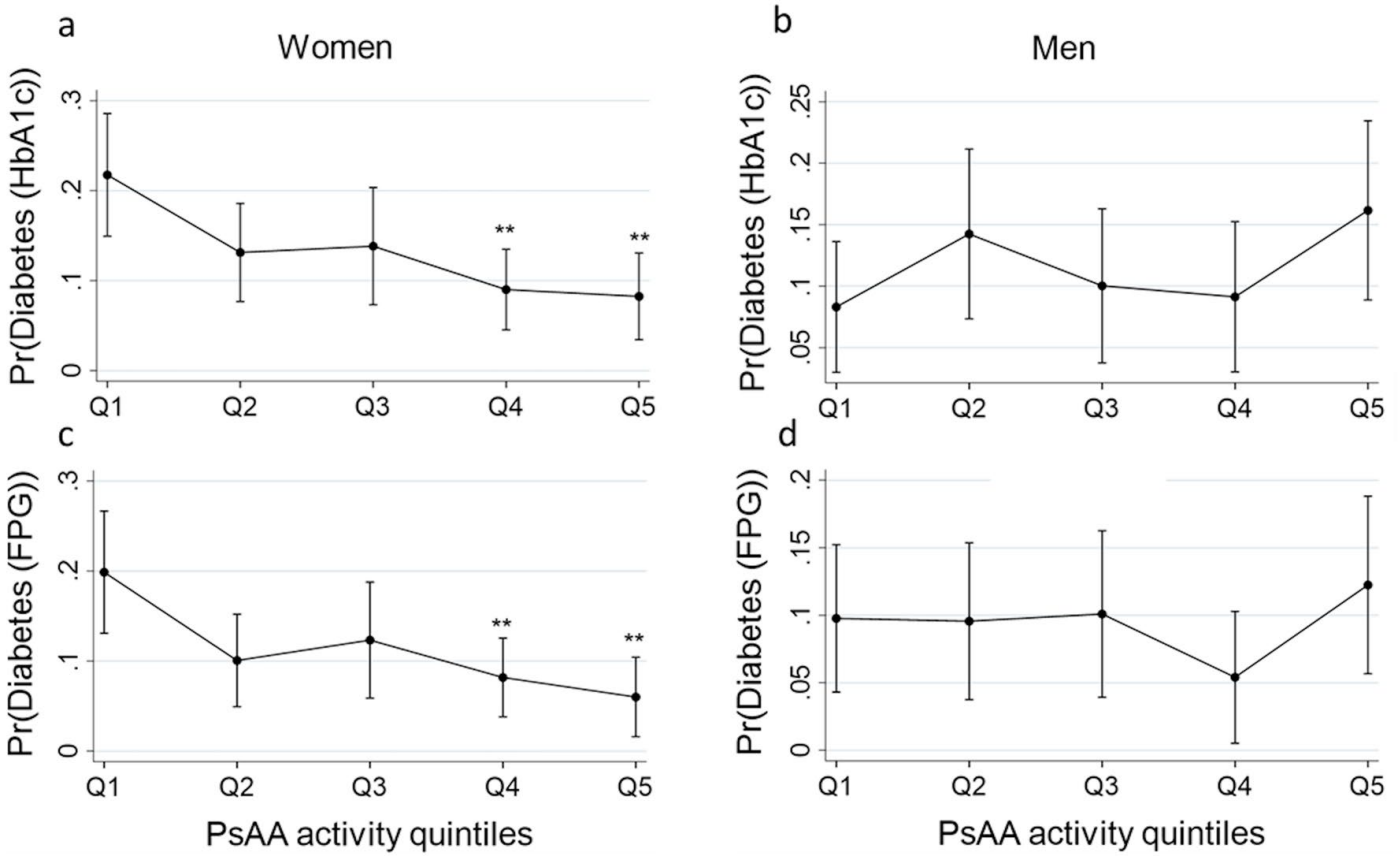
Predicted probabilities for having diabetes across pSAA activity quintiles by gender. Diabetes is defined with HbA_1c_ (**c,d**) or FPG (**a,c**). **Indicate significant difference (1% level) relative to Q1. The bars represent 95% confidence interval.

Afterwards, we asked whether or not the significant effect of pSAA activity on the odds of having diabetes was homogeneous through the distribution of pSAA activity within the study group. Hence, we reexamined the association between pSAA activity and odds of having diabetes across the pSAA activity quintiles (Q1 to Q5) in men and women. The results show that it is mostly the higher levels of pSAA activity that drive the association between pSAA activity and the reduced odds of having diabetes we observed in women (Table 3). Figure 2a shows that in women, the predicted probabilities of having diabetes, calculated based on the adjusted logistic regression equation, decrease significantly between the first quintile Q1 and the two last quintiles Q4 and Q5 (e.g. *pr* = 21.7% ± 0.03% in Q1 and *pr* = 8.2% ± 0.02% in Q5, when we use HbA_1c_ cutoff to define diabetes). Similar results are observed with FPG cutoff (Fig. 2c). However, no significant decrease in probability was found in men (Fig. 2b,d). To corroborate our finding, we compared the glycemic parameters HOMA-IR, HOMA-β, FPG, HbA_1c_, and basal insulin between Q1 and Q5 of pSAA activity in women (Supplementary Table S1). The results indicate that the mean HOMA-IR is significantly different between Q1 and Q5 (3.4 ± 3 (Q1) versus 2.5 ± 2.3 (Q5) p = 0.02). Interestingly, the mean HOMA-IR in Q1 is higher, while the mean HOMA-IR in Q5 is lower than 2.9, the cutoff set to indicate insulin resistance, suggesting that women in Q1 are insulin resistant. There was no significant difference in HOMA-β between Q1 and Q5, although it was higher in Q5 (Supplementary Table S1). Moreover, both FPG and HbA_1c_ were significantly higher in Q1 as expected (Supplementary Table S1). Lastly, we found that the prevalence of diabetes is significantly higher in the Q1 as compared to Q5 in women (Fig. 1h, Supplementary Table S1). Further, the mean pSAA activity was significantly lower in women with diabetes (Fig. 1e,f).

**Table 3:** Odds of having diabetes across pSAA activity quintiles using logistic regression adjusted for age and BMI.

	Men					Women				
	Q1 (87)	Q2 (86)	Q3 (86)	Q5 (86)	Q5 (86)	Q1 (100)	Q2 (100)	Q3 (99)	Q4 (100)	Q5 (99)
	OR (95% CI)					OR (95% CI)				
Diabetes (HbA_1c_)	1	1.96 (0.72–5.33)	1.25 (0.42–3.71)	1.11 (0.36–3.42)	2.33 (0.86–6.29)	1	0.41 (0.17–1.01)	0.45 (0.17–1.18)	0. 23 (0.09–0.61)**	0.21 (0.74–0.60)**
Diabetes (FPG)	1	0.97 (0.34–2.75)	1.04 (0.36–2.98)	0.49 (0.13–1.73)	1.34 (0.48–3.72)	1	0.34 (0.13–0.88)*	0.46 (0.17–1.22)	0.26 (0.10–0.67)**	0.17 (0.003–0.58)**

Diabetes was defined either with HbA1_c_ or FPG cutoff.

The **indicate significance (1% level) relative to reference quintile, i.e. Q1.

*p < 0.05, **p < 0.01, ***p < 0.001

## Discussion

This study shows a significant association of high levels pSAA activity, but not AMY1 CNV, with lower odds of having diabetes exclusively in adult Qatari women (OR per pSAA activity unit 0.95 [95% CI 0.92, 0.98] (p = 0.002); with pSAASAA activity varying from 4.7 to 65 U/L in the population). The inverse association found remained significant regardless of age and BMI. However, we have found that the observed global effect of pSAA activity on the odds of having diabetes is primarily due to the contribution of the highest levels of pSAASAA activity. As a result, the diabetes prevalence decreased significantly from the first (Q1) to the fifth (Q5) pSAA activity quintile (Fig. 1h). In addition, we found that women in Q1 are significantly more resistant to insulin, as estimated by HOMA-IR, than women in Q5, which is likely to explain the high prevalence of diabetes in Q1. β-cell function, as estimated by HOMA-β, was also lower in Q1, although not significantly. Our results are in line with a previous work^25^, which reported that pSAA activity, but not AMY1 CNV, was significantly associated with lower fasting plasma glucose levels in a French study of 3500 adults. Other studies have also suggested that high pSAA activity is associated with improved tolerance for glucose after starch load^23,24^. In contrast to us, a recent study^22^ has found that low AMY CNV is associated with unfavorable glucose metabolism. We have no explanation for this discrepancy, but we note that only young (20–39 years), non-obese women were enrolled in that study.

A number of studies have shown a positive correlation between AMY1 CNV and plasma pSAA expression^3,26^ and activity^26,27^. There was also a positive correlation between AMY1 CNV and pSAA activity in our population (r = 0.44, p < 0.001), which explained just 20% of the variance in pSAAactivity compared to 27% and 38% in Mandel and Atkinson studies, respectively^26,27^. This observation indicates that, as suggested earlier^6^, copy number variation of the human AMY1 gene is not the only factor that determines the variability in pSAA expression and activity. Other factors, including diet, stress levels and circadian rhythms, are likely to influence SAA production and activity^2^. Positive or negative effects of single nucleotide polymorphisms (SNPs) within or near AMY1 locus on the expression and the SAA activity cannot be excluded either and warrants further investigation^28,29^.

Our findings raise the question of the generalizability of the effect of AMY1 CNV on metabolism even in the same population, i.e. two individuals may have the same AMY1 CNV but significantly different pSAA activities resulting in opposite effects on carbohydrate metabolism and glucose handling. Therefore, extra copies of the AMY1 gene may not necessarily mean increased capacity to digest starch. Caution must thus be exercised when examining the influence of AMY1 CNV and potentially other genes with CNV in this regard. Our findings indicate that knowing the AMY1 CNV is not sufficient to predict the predisposition to glucose intolerance or obesity^5^ without knowing how much active pSAA is produced. The SAA is the end-product of the AMY1 gene and is essentially the factor that determines the rate of starch degradation and, therefore, the amount of sugar produced. As a result, we believe that pSAA activity is the most suitable parameter for evaluating the relationship between SAA and metabolic disorders, such as glucose intolerance, obesity or insulin resistance. In addition, unlike AMY1 CNV, the quantification of which resulted in conflicting results^5,16,25^, the SAA activity has the advantage of being easy to quantify reproducibly. Moreover, with AMY1 CNV one can only predict the SAA activity, while the SAA activity measurement reflects the actual capacity to degrade the carbohydrates.

Our observation that the effect of pSAA activity on the occurrence of diabetes is detected only in women also highlights the importance of gender-specific data analysis because of the effect of hormones and other factors on metabolism. It further raises the question of the generalizability of the associations reported previously between AMY1 CNV and the different metabolic disorders even within the same population. The lack of effect of pSAA activity in men warrants further investigation to better unravel the underlying mechanisms.

The distribution of AMY1 gene CNV in our study sample (2–20 copies) is comparable to what was reported previously across modern human populations^30^. The median of AMY1 gene CNV was 7 in our sample population, which is similar to the one reported in a recent study^10^. However, other studies previously reported a median of 4^5,20^. We have no explanation for this large difference in median AMY1 CNV, but the different age, ethnicities studied, or the techniques used to estimate the AMY1 CNV might play a role.

Although the mean AMY1 CNV did not differ between sexes in our population, the pSAA activity was significantly higher in men (Table 1). This observation suggests a possible sexual dimorphism of AMY1 gene expression and/or translation, i.e. some AMY1 gene copies may be less expressed/translated in women or more expressed/translated in men, with functional consequences to utilization of carbohydrates by men and women. Furthermore, we found that the mean pSAA activity, but not the mean AMY1 CNV, is significantly lower in women with diabetes, corroborating the effect of pSAA activity, and not AMY1 CN, on odds of having diabetes.

The mechanisms that link pSAA activity to odds of having diabetes remain elusive. Recent studies have shown that basal saliva SAA activity is inversely associated with behavioral preference for foods high in sugar^21^. Consequently, in regions, such as the Middle East, where diets are rich in starch, women with low pSAA activity might be at higher risk of weight gain and diabetes because of increased intake of sugary foods. Previously, Mandel et al.^23^ showed that normal-weight adults with low SAA activity displayed glucose intolerance upon liquid starch ingestion and suggested the lack of early pre-absorptive insulin secretion as the underlying mechanism. Baling et al.^24^ have advanced that the improved glucose response observed in subjects with high SAA activity following a starch bolus might be explained by the stimulation in the proximal part of the intestine of a rapid secretion of the gastric inhibitory peptide (GIP), which in turn stimulate insulin secretion from the pancreatic beta cell. Furthermore, low quantities of saccharides, such as maltose, produced in low amylase individuals may not be detected by the sweetness receptors in upper sections of the gastrointestinal tract. Consequently, the coupling of sweet taste with the release of a number of glucose metabolism and appetite-associated signaling peptides such as GLP-1 and PYY might be broken, and hence alter the CNS-mediated regulation of insulin secretion for example^31^. Given the link between obesity and T2D, the results of the present study along with the findings form other studies reinforce the hypothesis that high SAA activity is potentially beneficial for energy metabolism and glucose tolerance mainly in populations with diets rich in starch, like in the Middle East region.

Further, given the role of SAA in the digestion of starch, variation in this enzyme’s activity might influence gut microbiota through dietary carbohydrate processing^9,32^. Lately, low AMY1 CNN was associated with gut *Prevotella* abundance in Mexican children and adults^9^. *Prevotella* was previously reported to be metabolically favorable^33^. Additionally, an association was reported between AMY1 activity and lactate, a product of complex intestinal carbohydrate fermentation^25^. Further investigations are needed to fully understand how SAA activity might contribute to the modulation of the function of the gastrointestinal tract in humans and directly or indirectly affect glucose homeostasis.

The main limitation of our study is that it is cross-sectional and not prospective. Therefore, we can only report associations. Additionally, our analyses did not include adjustment for lifestyle factors such as smoking status, exercising, alcohol consumption, and diet, which could interfere with the results of the regression analysis and correct the effects of abundant carbohydrate diet, sedentary lifestyle and smoking habits. On the other hand, this is the first study that investigated the relationship between SAA and diabetes in the Middle East, where diabetes has reached epidemic proportions. Identification of markers to predict the predisposition to diabetes is of paramount importance for the fight to curb the diabetes epidemic in the region.

## Conclusions

In summary, there was a significant association between pSAA, but not AMY1 CN, and reduced odds of having diabetes in adult Qatari women. This association is primarily driven by high levels of pSAA activity. Our finding, along with the results from other studies are suggestive of a potential benefit of high salivary α-amylase activity on carbohydrate metabolism and glucose homeostasis, especially in women in the Middle East in general, and in Qatar in particular, where starch is a staple food. The mechanisms underlying the beneficial effect on reduced diabetes odds remain to be elucidated.

## Research design and methods

### Study design and participants

For this cross-sectional study, we used plasma as well as clinical, anthropometric, and demographic data of 929 adults from the Qatar Biobank (QBB). Participants were included in this study if they were Qatari nationals, have been fasting more than 6 h, and their whole genome sequencing data were available. We did not use any exclusion criteria. QBB is a cohort established for research purposes and enrolls adults (aged > 18 years), who are either Qatari citizens or long-term residents (residing in Qatar for at least 15 years) from the general population^34,35^. The present study was approved by the the institutional review boards at both Qatar Biomedical Research Institute (QBRI) (IRB number: 2017-001) and QBB (IRB number: Ex-2017-RES-ACC-0054-0018). All work was performed in compliance with the ethical standards stated by the declaration of Helsinki. All participants gave informed written consent for the use of their data and biospecimens in medical research.

### Anthropometric and clinical measures

Plasma samples were prepared according to a standard protocol within 2 h of collecting the blood. All the blood biochemistry measurements were performed at the central laboratories at the Hamad Medical Corporation in Doha. In this paper, we define diabetes based on the recommendations of the American Diabetes Association. A person has diabetes if the HbA_1c_% is ≥ 6.5% (≥ 48 mmol/mol) or the fasting plasma glucose level (FPG) is ≥ 126 mg/dL (≥ 7 mmol/L). We calculated HbA_1c_ in mmol/mol from HbA_1c_% using the formula: (HbA_1c_ × 10.93) − 23.5. Body mass index (BMI) was calculated as weight in kilograms divided by height in meters squared (kg/m^2^). We used Caucasian cutoffs for categorization of BMI (normal weight: 18 ≤ BMI < 25; overweight: 25 ≤ BMI < 30; obese BMI ≥ 30 kg/m^2^). For the assessment of insulin resistance, we used the Homeostasis Model Assessment of insulin resistance (HOMA-IR) equation; HOMA-IR = (fasting insulin (µU/L) × fasting glucose (mmol/L))/22.5. For the assessment of the pancreatic beta-cell function we used the Homeostasis Model Assessment b equation (HOMA-β); HOMA-β = ((20 × fasting insulin (µU/L))/(fasting glucose (mmol/L) − 3.5)) × 100.

### Quantification of the salivary α-amylase activity in plasma

The plasma SAA activity (pSAA activity) was quantified using an enzymatic colorimetric assay with an autoanalyzer (ARCHITECT c4000; kits # 6K22-30 and # 7D58-21; ABBOTT laboratories, Bluff, Illinois, USA) according to manufacturer's protocol. Details of the assay are reported in supplementary material.

### Estimation of AMY1 CNVs

We estimated the AMY1 gene CNV from whole-genome sequencing data using the version 0.4 of CNVnator, which uses read-depth (RD) genome sequencing analysis for CNV discovery and genotyping^36^. Briefly, out of the WGS bam files, Chr1-aligned reads were extracted and indexed using Bamtools^37^. Read mappings were collected, and the RDs were determined using CNVnator for regions of 1 kb. The reads were partitioned into 100 base regions that were not overlapping and were instead normalized. CNV counts were then calculated from the uniform read counts and filtered for interest regions. Test CNV counts were then calculated by summing the counts for the regions of interest unique to AMY1A. We rounded the copy number of AMY1 to the nearest integer.

### Statistical analysis

All statistical analyzes were carried out using Stata 15.1/IC software (http://www.stata.com). Descriptive statistics were used to present the mean and standard deviation, median, and proportion data. The comparison of continuous characteristics between groups was performed with unpaired t-test for independent samples. For proportions, we used the Chi^2^ test. Variables with outliers were winsorized using winsor2 command in Stata. Associations were assessed with the Pearson correlation coefficient and linear regression. Adjusted logistic regression was used to assess the association of pSAA activity or AMY1 CNV with the odds of having diabetes. When we run logistic regression across the pSAA activity quintiles, the first quintile is considered the base level. None of the variables had more than 7% missing values, which were imputed using multiple imputations by chained equations in Stata. A *p*-value < 0.05 was considered statistically significant.

### Ethical approval

The present study was approved by the institutional review boards at both Qatar Biomedical Research Institute (IRB number: 2017-001) and Qatar Biobank (IRB number: Ex-2017-RES-ACC-0054-0018). All participants gave written informed consent for their data and biospecimens to be used in medical research.

## Supplementary Information

Supplementary Information.

## Data Availability

Clinical, anthropometric, demographic and genetic data can be obtained from the Qatar biobank according to the applied rules.
