# Supplementary material for: Reduced odds of diabetes associated with high plasma salivary α-amylase activity in Qatari women: a cross-sectional study
Source: Sci Rep. 2021 Jun 1;11:11495. doi: 10.1038/s41598-021-90977-y (PMC8169920; doi:10.1038/s41598-021-90977-y)
Supplement: Supplementary file 1 — Supplementary Information. [file 41598_2021_90977_MOESM1_ESM.docx]

## Reduced odds of diabetes associated with high plasma salivary α-amylase activity in Qatari women: a cross-sectional study

## Neyla S Al-Akl^1^, Richard Ian Thompson^1^, Abdelilah Arredouani^1,2,*^

**Supplementary material**

**Methods**

Quantification of plasma sAA activity

The quantification of the plasma sAA activity (psAAa) was estimated by an enzymatic colorimetric assay with an autoanalyzer (ARCHITECT c4000; kits # 6K22-30 and #7D58-21; ABBOTT laboratories, Bluff, Illinoi, USA). The procedure consists of two reactions to measure the enzymatic activities of the total α-amylase (tAA) and the pancreatic α-amylase (pAA). The activity of the sAA is obtained by subtracting the activity of the pAA from that of the tAA. To obtain the pAA activity the assay is performed in two successive steps (kit # 6K22-30). In the first step, the activity of the sAA is inhibited using two different monoclonal antibodies with no effect on the pAA. In the second reaction step, the pAA catalyzes the hydrolysis of the EPS substrate (Ethydien Protected Substrate) *p*-*Nitrophenyl*-*maltoheptaoside 4,6-ethylidene-blocked (ethylidene-G7PNP) forming 2 ethylidene-G5 + 2 G2PNP + 2 ethylidene-G4 + 2 G3PNP + ethylidene-G3 + G4PNP. The α-glycosidase hydrolyzes all the fragments of the G2PNP, G3PNP, and G4PNP into phenol (PNP) and glucose (G). The increase of absorbance due to PNP formation is proportional to the activity of pAA in the examined sample. For the tAA activity,* α-amylase hydrolyzes the 2-chloro-4-nitrophenyl-α-D-maltotrioside (CNPG3) substrate to release 2-chloro-4-nitrophenol (CPNP) and form 2-chloro-4-nitrophenyl-α-D-maltoside (CNPG2), maltotriose, and glucose. The rate of formation of the 2-chloro-4-nitrophenol can be detected spectrophotometrically at 404 nm to give a direct measurement of the total α-amylase activity in the sample. Plasma sAA enzymatic activity was calculated by subtracting the activity of the pAA from the activity of tAA (psAA = tAA-pAA).

**Results**

| Table S1. Comparison of baseline characteristic of women across psAAa quintiles | | | | | |
| --- | --- | --- | --- | --- | --- |
|  | Women | | | | |
|  | Q1(100) | Q2 (100) | Q3(99) | Q4 (100) | Q5 (99) |
| Age (years) | 39 (13) | 41 (12) | 38 (12) | 43 (13) | 39 (15) |
| AMY1 CNV (median) | 5 | 7 | 7 | 8 | 10 |
| AMY1 CN (mean) | 6 (3) | 7 (2) | 8 (3) | 8 (3) | 10 (3) |
| pSAAs (U/L) | 14 (4) | 22 (2) | 29(2) | 36(3) | 49 (7) |
| Obesity % | 53 | 51 | 37 | 56 | 34* |
| Overweight % | 27 | 27 | 38.3 | 29 | 33.3 |
| Diabetes % (HbA1c) | 20 | 13 | 10* | 12 | 9 * |
| Diabetes % (FPG) | 19 | 10 | 9* | 11 | 6** |
| BMI (Kg/m2) | 30.51 ±6.67 | 29.77 ±6.47 | 28.71 ±6.02 | 30.91 ±5.99 | 28 ± 6.17 |
| FGP (mmol/l) | 6.2 (2.7) | 5.5 (1.3) * | 5.5 (1.6) * | 5.5 (1.1) * | 5.4 (1.5) ** |
| HbA1c (%) | 6.0 (1.3) | 5.7 (1) * | 5.6 (0.9 ) * | 5.8 (0.8) | 5.6 (0.8) * |
| Insulin (mU/ml) | 11.9 (8.3) | 10.5 (6.7) | 9.9 (5.4) * | 11.5 (7.5) | 9.8 (7.2) |
| HOMA-IR | 3.4 (3) | 2.7 (2.2) | 2.5 (1.9) * | 2.9 (2) | 2.5 (2.3) * |
| HOMA-B | 114 (66) | 120 (64) | 116 (56) | 129 (84) | 119 (80) |

**p < 0.01 versus Q1

*p < 0.05 versus Q1
